# Retrospective analysis of Vogt-Koyanagi-Harada’s recurrence within a case series of nineteen patients followed between 2003 and 2018 in an academic center

**DOI:** 10.1186/s12348-025-00498-2

**Published:** 2025-07-01

**Authors:** Alexandra Kozyreff, Alice Bughin, Chantal Lefebvre, Lucie Pothen, Halil Yildiz

**Affiliations:** 1 Cliniques universitaires Saint-Luc, Catholic University of Louvain, Brussels, Belgium; 2 Uveitis Department, Cliniques universitaires Saint-Luc, Av. Hippocrate 10, Brussels, 1200 Belgium

**Keywords:** Vogt-Koyanagi-Harada disease, Granulomatous choroiditis, Recurrence, Acute initial phase, Chronic recurrent phase, Treatment, Survival curve

## Abstract

**Purpose:**

To define a timeframe of recurrences according to the clinical presentation of inflammation and the course of the disease. This could influence final visual acuity and avoid ocular complication such as cataract, glaucoma, choroidal neovascularization, subretinal fibrosis or fundus depigmentation.

**Materials and methods:**

Retrospective study of nineteen patients affected by Vogt-Koyanagi-Harada disease followed between 2003 and 2018.

**Results:**

Within our case series, 53% of patients had no recurrence during a follow-up of up to one hundred and sixty-eight months. Among the nine patients with recurrence, five had at least one episode of posterior inflammation, one exhibited both anterior and posterior recurrence, and five developed at least one recurrence in anterior structures. According to the Kaplan Meier method, the overall recurrence-free survival at three months is 68% ± 11%. All posterior segment inflammatory relapses occurred within three months and a half of systemic treatment initiation. On the other hand, the timeline of anterior recurrence is more scattered. They occurred between two and thirty-seven months of treatment initiation. This careful follow-up, which differentiated between types of inflammatory recurrences, made it possible to observe final visual acuity greater than or equal to 9/10 (equivalent ≥ 20/25), with the exception of one amblyopic eye.

**Conclusion:**

The chronological occurrence of posterior inflammatory episodes is earlier than anteriorly. A posterior recurrence does not increase the risk of anterior inflammation relapse during the follow-up. This distinction is important to understand the course of the disease. In fact, the differentiation between the initial acute phase (mainly posterior inflammations) and chronic recurrences (granulomatous anterior segment inflammation) allows for better adaptation of systemic therapy and better visual prognosis in the long term.

## Background

The aim of our study is to present inflammatory recurrences according to their chronology. Vogt-Koyanagi-Harada (VKH) disease is a posterior granulomatous uveitis involving the choroid associated or not to a panuveitis spillover. Several hypotheses of VKH’s pathogenesis stem from a maladaptive T-mediated immune response against melanocytes’ self-antigen [1]. The inflammation affects uvea, stria vascularis of the cochlea, meningeal structures and the skin. It predominates in more pigmented populations such as Asians, Hispanics, Afro-Americans or Indians [2]. In the United States of America, 3–4% of uveitic patients seen in tertiary centers are affected by VKH disease [2]; whereas in Japan, this number can rise up to 9% depending on series [3]. In Belgium, university hospitals receive an average of one or two cases per year in each hospital.

**Fig. 1:**
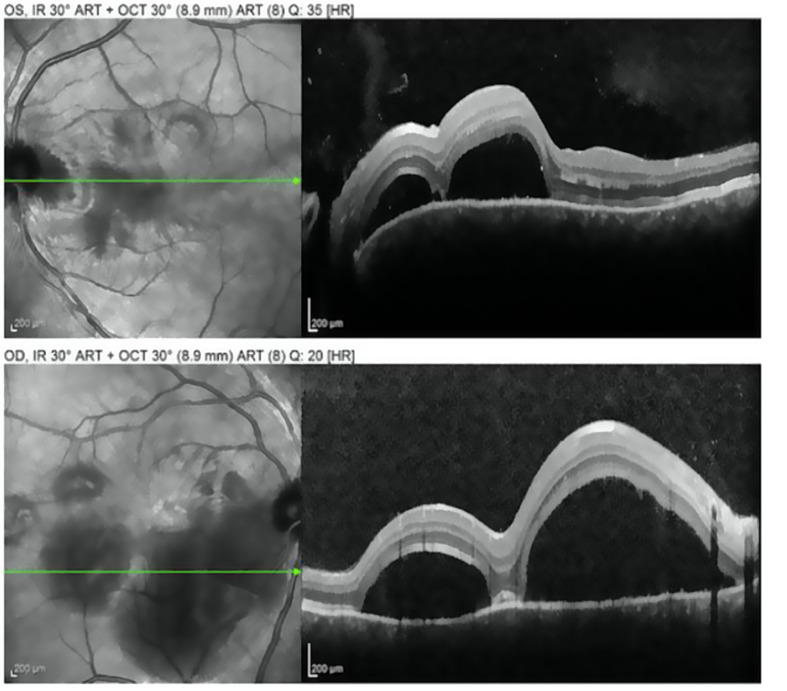
OCT of a patient suffering from VKH disease, followed at CUSL, and showing multilobed serous retinal detachment (acute uveitis)

The clinical course of the disease was divided into four stages: prodromal (neurological, auditory and flu-like symptoms); acute uveitis (acute bilateral granulomatous panuveitis with typical “dome-shaped ‘’ serous retinal detachments, as illustrated in Fig. 1, associated or not with papillitis); chronic uveitis (limbic or fundus depigmentation (Sugiura’s sign or sunset glow fundus)); chronic recurrent (anterior segment inflammation) [4]. This understanding is important from an ophthalmological point of view because it differentiates the occurrence of posterior (worsening of papillitis, choroidal thickness, serous retinal detachment, vitritis) and anterior (anterior chamber flare, iridocyclitis, white keratic precipitate, posterior synechia) segment relapses. One of the objectives of our study is to emphasize the recognition of acute uveitis compared to the chronic relapsing phase of the disease. This distinction has a major impact on final visual acuity as it reduces sight-threatening complications such as cataract, glaucoma, choroidal neovascularization, subretinal fibrosis or retinal scars [5].

The revised diagnostic criteria were established by the American Association of Ophthalmology (AAO) in 2001 depending on the multi-system damages. According to their classification, the disease is categorized as “probable” (if only ocular signs are present), “incomplete” (if ocular combined with neurological and/or integumentary findings are present) or “complete” (if early and late ocular, neurological and cutaneous manifestations are present) [6]. However, several clinicians have pointed out certain limitations of these criteria as they do not take into account the course of the disease or more precise ancillary tests such as ICG angiography, EDI-OCT or SS-OCT [7–9]. In addition, the “complete” form is incongruous at the initial stage of the disease because the skin manifestation appear weeks later, in the chronic phase. In 2018, the Standardization of Uveitis Nomenclature (SUN) reported a moderate agreement rate (mean kappa 0,4) among uveitis experts on VKH revised diagnostic criteria [10]. The main objective of our study is to emphasize the value of a classification based on the initial acute inflammatory phase and chronic recurrent anterior segment inflammation.

Our case series spans fifteen years. Therefore, the therapeutic modalities have changed over the years. At the beginning of the series, four patients received corticosteroid therapy with intravenous bolus relayed by tapering dose of oral methylprednisolone over a minimum of 18 months. Subsequently, the patients were treated with a combination of corticosteroid and an immunosuppressive agent to avoid systemic complications due to long term corticotherapy.

Our study will also show that understanding the course of the disease will guide the treatment. For the acute panuveitic phase, the current therapeutic guidelines recommend three intravenous boluses of 1 gram of methylprednisolone followed by oral 0.5 to 1 mg/kg/day on a decreasing basis, in conjunction with immunosuppressants (Azathioprine (2–3 mg/kg/day), Methotrexate (maximum 25 mg/week), mycophenolate mofetil (2000 g daily), Cyclosporine (2.5-3 (maximum 5) mg/kg daily,)) [11]. In case of insufficient response, biotherapies such as anti-TNF α can be proposed [11, 12]. This therapeutic approach was applied to fifteen patients in our series. However, a topical management should be preferred in the event of chronic anterior of inflammation.

**Fig. 2:**
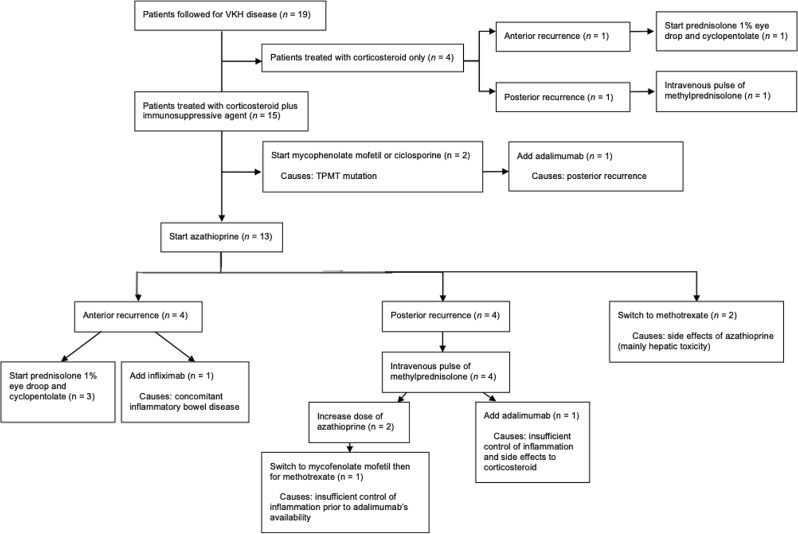
VKH disease treatment regimen flowchart at CUSL

Our therapeutic attitude is depicted as a flowchart in Fig. 2.

### Main text

#### Study design

Our study is a retrospective descriptive analysis of patients diagnosed with Vogt-Koyanagi-Harada disease. All patients were followed at Saint Luc University Hospital between 2003 and 2018.

The ethics commission of our hospital approved the study (reference 2018/25AVR/186) andthe authors declared no financial interest or bias with pharmaceutical companies.

#### Patient enrollment

All patients diagnosed according to the revised diagnostic criteria for Vogt-Koyanagi-Harada disease were eligible for the study. Some of them presented at our hospital immediately after the onset of symptoms while others were then referred to our university center.

The patients were enrolled regardless of their age, sex or ethnicity. The proportion of ethnic representation in our series should be understood with the history of Belgian migration flows [13] and the prevalence of the disease among patients with more pigmented skin.

None of the patients included had a history of intraocular trauma or surgery. This parameter allowed us to exclude cases of sympathetic ophthalmia; which has clinical manifestations and angiographic patterns similar to Vogt-Koyanagi-Harada disease [14].

#### Data collection method

Data points were collected from the patient’s medical files of the patient and were then anonymized for the analysis.

The same diagnostic devices have been used to produce images such as OCT or FA and ICGA combined.

#### Statistical method

Continued values in the subsequent tables were calculated with averages according to the standard deviation and medians with their ranges. On the other hand, discontinued values were summarized according to their occurrence as apercentage.

Graphics were produced by computer softwares such as Excel and SPSS.

#### Results

**Fig. 3:**
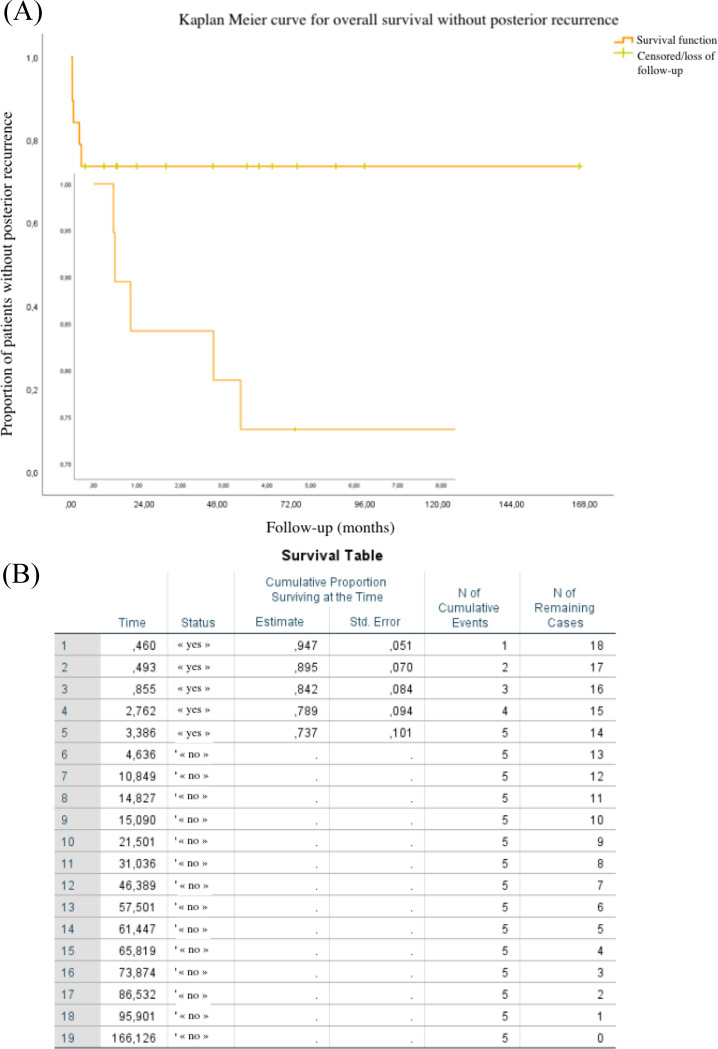
survival curve (**A**) and table (**B**) of patients without any posterior recurrence according to Kaplan Meier method

In our case series, Vogt-Koyanagi-Harada disease prevails among women at the mean age of forty-two (compared to a reported average of thirty-five years in the literature [4]). Conversely, 12% of our sample are male, with an average age of thirty-three (see Fig. 3).

The diagnosis of the disease was based on a clinical evaluation and according to imaging criteria described in the literature [6, 15, 16] (on EDI-OCT, on fluorescein and indocyanine green angiographies images). In the acute stage of the disease, seven patients of our series underwent a lumbar puncture, confirming a lymphocytic meningitis. The characteristics of initial clinical presentations are listed in Tables 1 and 2 below.

**Table 1:**
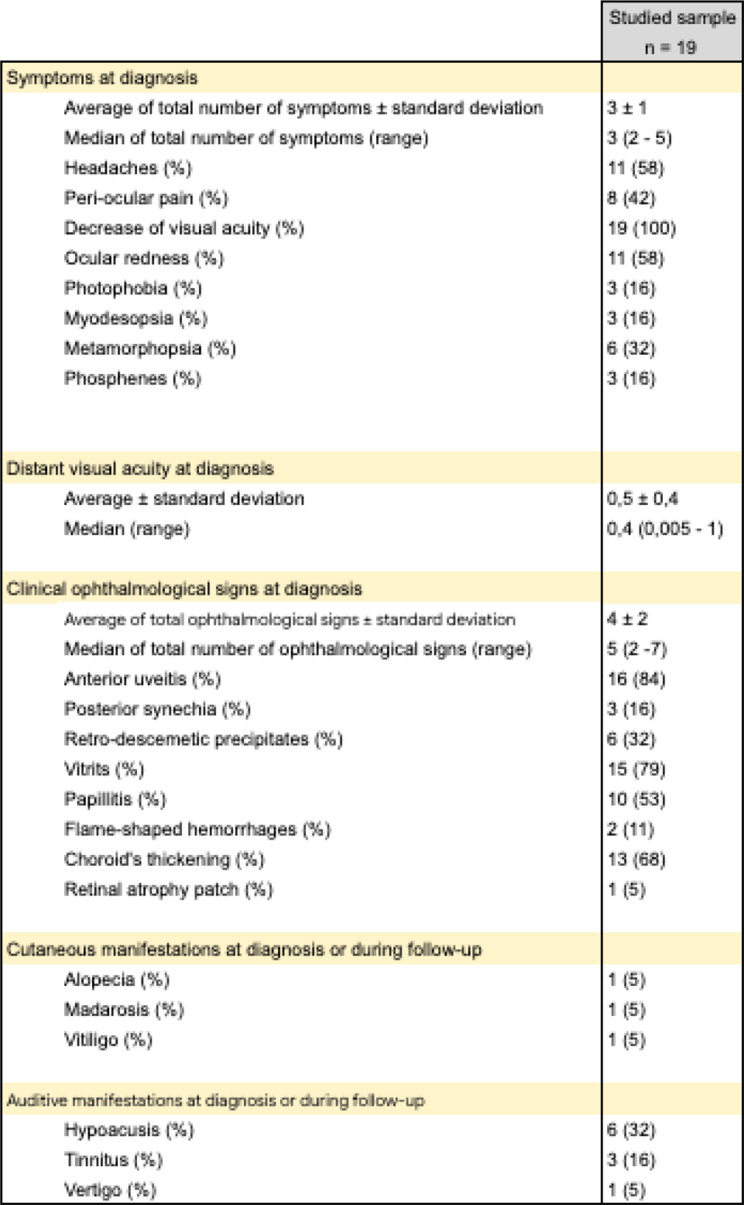
Initial clinical manifestations of patients followed at saint Luc university hospital

**Table 2:**
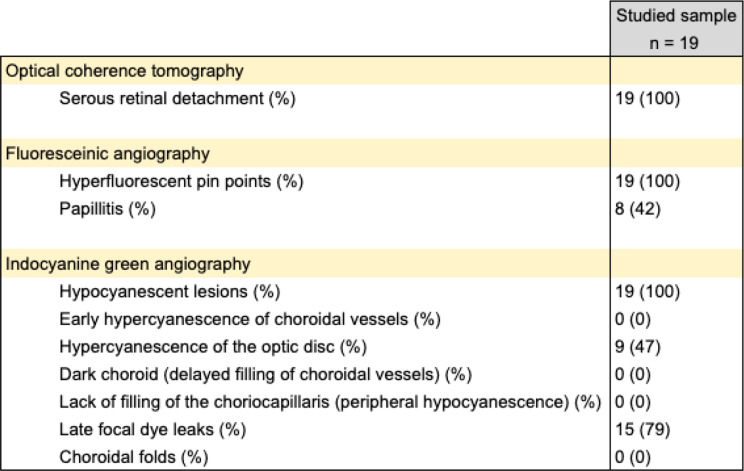
Characteristics of imaging during presentation at saint Luc university hospital

The median diagnostic delay for VKH cases in our hospital is fourteen days. Due to this early diagnosis, none of the patients in our series developed a complete clinical presentation as defined by the American Association of Ophthalmology.

The occurrence of recurrences was classified according to their location (inflammation of the anterior or posterior segment) and their timeline (precocious if within three months after initiation of a systemic treatment and late if beyond three months)). Kaplan Meier method produces survival curves that represent the proportion of patients without any inflammatory recurrence, month by month. Each downward horizontal step represents a patient who had relapsed. This statistical method also takes into account censored events such as stable patients who withdrew to pursue follow-up by their routine ophthalmologist.

In this study, 50% of all recurrences were posterior, which represents five relapsing patients. The criteria for posterior recurrences were clinically based on the reappearance of choroidal thickening with or without subretinal serous detachment or papilledema. These manifestations may or may not be associated with an anterior chamber reaction and keratic precipitates.

In this study, most cases (60%) experienced only one posterior recurrence. In contrast, one patient presented three posterior recurrences and subsequently manifested six episodes of anterior segment inflammation.

All posterior recurrences appeared between the fourteenth and the one hundred and third days after treatment initiation with methylprednisolone bolus.

According to the Kaplan Meier survival curve in Fig. 3A, fourteen patients (i.e. 74%) did not show any posterior recurrence during the follow-up (one hundred six months). After three months, three out of five patients had already presented a posterior recurrence. The Kaplan Meier table of Fig. 3B displays a 74% ± 10% non-recurrence rate of posterior inflammation beyond three months.

Anterior recurrences occurred in five studied patients (i.e. 26%). Clinical picture was categorized by anterior chamber reaction associated or not with conjunctival redness, posterior synechiae or keratic precipitates distant from the initial inflammation or from a posterior recurrence. All of them flared up between the fifty-sixth and one thousand one hundred twenty-seventh days, with a median of two hundred forty-seven days. Thus, the time to onset is much more variable for anterior recurrences compared to posterior ones. Among the 26% of patients with an anterior recurrence, the median occurrences of this type of inflammatory episodes was equal to two. This demonstrates an active chronic inflammatory process that is difficult to extinguish and highlights the importance of close follow-up to avoid long-term sequelae.

**Fig. 4:**
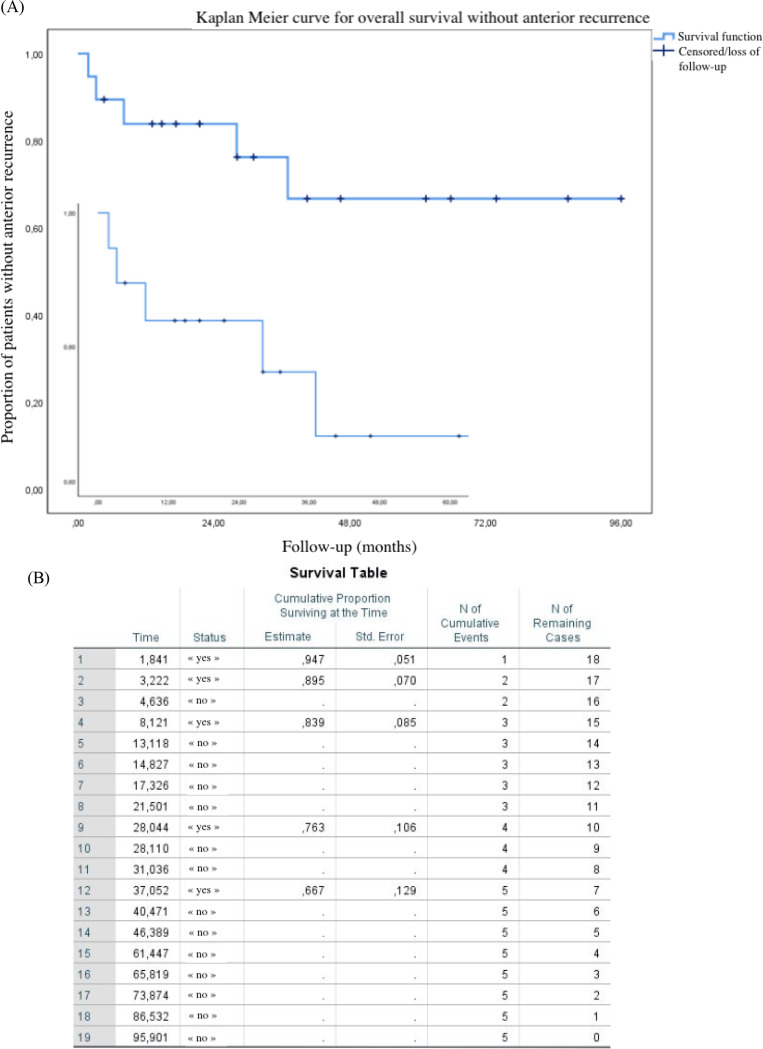
survival curve (**A**) and table (**B**) of patients without any anterior recurrence according to Kaplan Meier method

Based on the Kaplan Meier survival curve and table in Fig. 4, fourteen patients (i.e. 74%) showed no anterior recurrences and 84% ± 9% of patients are not expected to develop such recurrences after eight months.

**Fig. 5:**
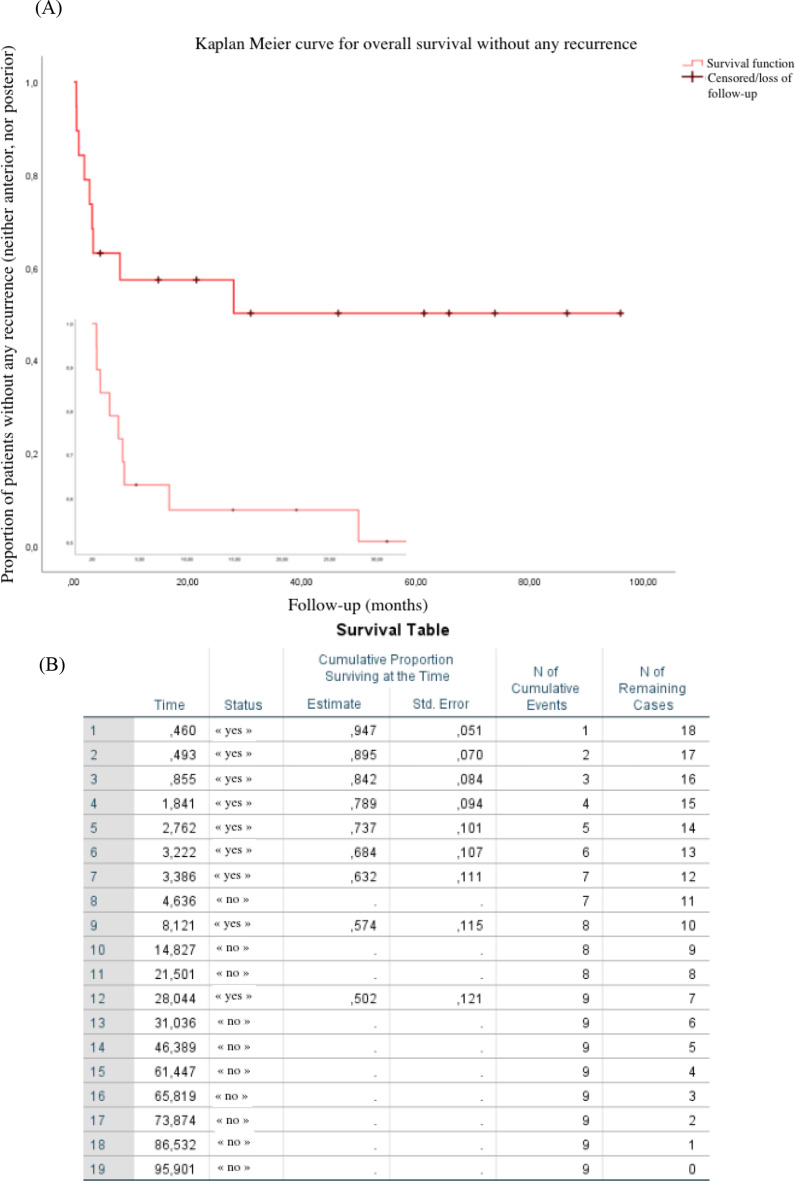
survival curve (**A**) and table (**B**) of patients without any recurrence according to Kaplan Meier method

Eleven studied cases (i.e. 58%) had no recurrence at all. Based on our data set, it is interesting to note that at three months, the Kaplan Meier method estimates the probability of patients with no inflammatory recurrence at 68% ± 11%. Fig. 5 It should also be noted that twenty-six days after initiation of bolus methylprednisolone treatment, one-third of recurrences had already occurred, and that these are exclusively posterior inflammatory reactions.

Posterior relapses therefore trend to occur earlier than anterior ones. This delay in onset is indicative of the initial site of inflammation, which is the choroid. Systemic treatments penetrate this vascular layer much better than other ocular structures. This underlines the importance of an early therapeutic window to control this posterior inflammation. In the case of a subsequent recurrence, the adjustment of systemic treatments has a key role.

In contrast, anterior inflammatory recurrences occur later, at a chronic stage of the disease. The inflammation is still present and affects the anterior structures. Treatment should be based on an adjustment of systemic immunosuppressive drugs and eye drops.

Over the long term, all patients followed at CUSL recovered an excellent final visual acuity of greater than or equal to 9/10 (equivalent to ≥ 20/25), with the exception of one amblyopic eye (left eye of patient 6 in Table 3).

**Table 3:**
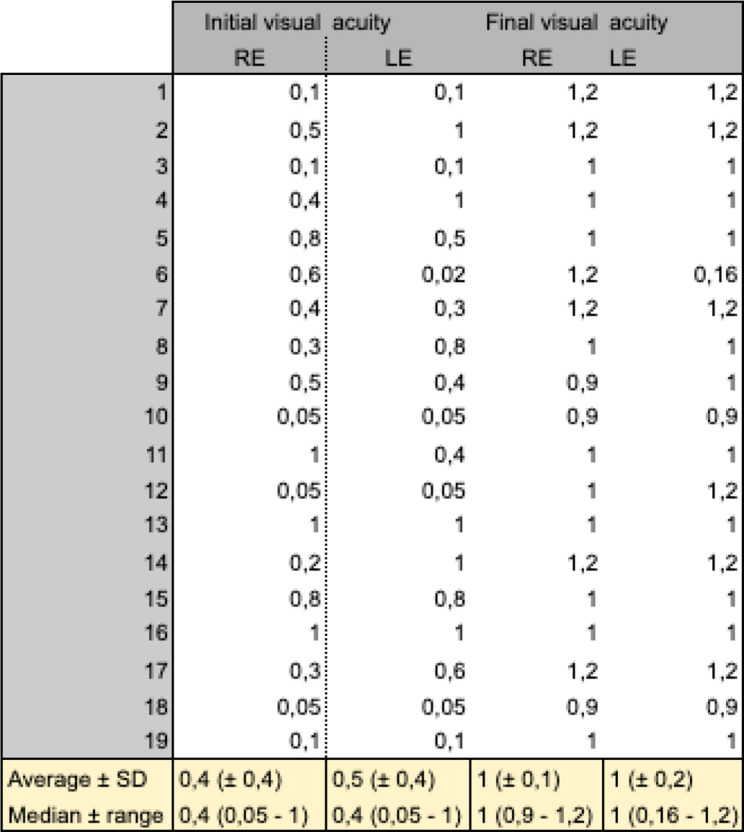
Visual acuity at initial and final study follow-up

The absence of ophthalmological complications such as cataract, glaucoma, subretinal fibrosis or choroidal neovascularization may explain the good final visual results in our series. Both the average and median of visual acuity are equal to 1,0 at the last follow-up.

Of the eight patients studied who developed inflammatory recurrences, indicating inadequate control of the disease, none of them developed post-inflammatory sequelae (beyond peripheral nummular scaring and migration of retinal pigmentary epithelium). Close monitoring, rapid therapeutic adaptation and a tapering regimen over almost eighteen months could prevent this type of event in our series. It is also worth highlighting the importance of early diagnosis (within two weeks) in order to quickly initiate an aggressive treatment with three intravenous pulses of methylprednisolone followed byan oral relay.

Similarly, the convalescence phase, defined by the appearance of a sunset glow fundus or depigmentation of the limbus (Sugiura’s sign), was not observed in any patient in the series despite follow-up over several years.

## Discussion

The results show that posterior recurrences occur earlier than anterior recurrences. In our study, all posterior recurrences occurred within one hundred and five days of the start of systemic treatment, whereas anterior recurrences were described up to more than one thousand days after the diagnosis. These observations are consistent with the distinctions made in the literature between the acute inflammatory phase (posterior uveitis or panuveitis) and chronic recurrences (anterior recurrences) [4]. The chronology of these posterior inflammatory recurrences underlines the choroidal origin of the inflammatory process. Interestingly, several studies reviewed subclinical choroidal inflammation, especially on ICG angiography, which may explain the late fundus depigmentation [17, 18]. This finding is consistent with sunset fundus histopathology showing disappearance of choroidal melanocytes and inflammatory T cell infiltrates [19].

Understanding this inflammatory process is important to adapt our therapeutic approach: initial posterior recurrences should be managed aggressively with boluses of methylprednisolone and a non-steroidal immunosuppressant, while anterior recurrences should be treated with an adjustment of immunosuppressive therapy and topical steroids. In case of refractory patients, biotherapies such as anti-TNF alpha monoclonal antibodies have been approved [11]. This distinction is important because it helps to avoid long-term complications as described in Ohno and al [5]: fundus depigmentation, cataract, glaucoma, subretinal fibrosis or choroidal neovascularization. In our series, none of our patients developed complications and no sunset glow fundus were observed, which can explain the good final visual results (mean and median equal to 1.0 (25/25)). All the eyes analysed in our series recovered visual acuity of 9/10 or better (≥ 20/25), with the exception of one amblyopic eye. In comparison, in the literature, a visual acuity ≥ 6/10 was obtained in two-thirds of cases in the study conducted by P. E. Rubsamen [20].

Furthermore, our study highlights the importance of an early treatment (within two weeks of the onset of the initial clinical disease) with high doses of corticosteroid and non-steroidal immunosuppressive agent to quickly annihilate the inflammatory process and avoid long term sight-threatening complications. Chee and al. also studied the role of a late diagnosis: early treatment (within two weeks) was associated with a better final visual acuity and fewer ocular complications than delayed diagnosis (between two and four weeks after the first symptoms) [21]. This therapeutic window and the crucial role of the combination between steroids and non-steroidal immunosuppressant were also described by Herbort and al [22].

The patients enrolled in our series were followed up in a tertiary university center, with parallel monitoring of systemic treatment by the internal medicine department of our hospital. This management enabled rapid correction of immunosuppressive treatments according to the inflammatory control of the disease and the occurrence of iatrogenic side-effects. The treatment regimen was based on Belgian recommendations for noninfectious uveitis [11].

However, this study has certain limitations. As Vogt-Koyanagi-Harada disease is a rare European condition (2% of uveitis cases recorded in three French reference centers, see page 2), our sample size is small compared to Japanese cohorts (a country where Vogt-Koyanagi-Harada disease may account for 6.8–9.2% of uveitis cases, depending on the series). As a result, patients enrolled between 2002 and 2018 did not receive the same treatment regimen (see line 114): some were treated with corticosteroids alone and others with a combination of cortisone and immunosuppressant.

In addition, the retrospective design of the study meant that we were dependent on data previously entered in the patient file. All clinical information was gathered by the same clinician. All FA and ICGA images were always taken by the same operator with the same Heidelberg angiography machine. Some patients were contacted again to obtain additional information for their follow-up.

As stated in the results, the analysis of recurrences was based on clinical data and not on subclinical signs of choroidal inflammation which would have been revealed on ICG angiography. For example, the study carried out by Dr. C. Herbort in Lausanne (Switzerland) has been able to highlight signs of persistent choroidal inflammation in a subclinical manner during regular angiographic follow-up with indocyanine green [7]. In Belgium, ICG dye is not reimbursed by social security and is quite expensive. Thus, in our study ICG angiography initially confirmed the diagnosis of choroidal inflammation but was not performed for therapeutic follow-up.

Other uveitis experts such as Urzua and al., have written on the interest of a distinction between the acute phase and chronic recurrences of VKH inflammation [23]. In fact, a Chinese study carried out in 2018 revealed that this differentiation refines the sensitivity to make the diagnosis (receiver operating characteristic curve (ROC) of 0,9 compared to 0,8 with the revised criteria) [24].

## Conclusion

Our study is a case series of nineteen patients enrolled in the university tertiary center of the Saint-Luc University Hospital (Brussels) between 2003 and 2018. Its retrospective design and long follow-up period allowed for an examination of the timeline of inflammatory flare-ups. Showing that posterior recurrences occurred more precociously than anterior ones. At three months, almost all inflammatory rebounds of the acute phase have taken place. Beyond three months, we could therefore define inflammatory recurrences as belonging to the chronic phase. The distinction is crucial for the management of treatments for patients with Vogt-Koyanagi-Harada disease and allows for a better long term visual prognosis. This analysis may also support a revision of diagnostic criteria and a standardization of the disease categorization.

## Data Availability

The datasets analysed during the current study are available from the corresponding author on reasonable request. The data used in this research are not openly available due to reasons of sensitivity.

## Abbreviations

AAO: American Association of Ophthalmology
Anti-TNF: Anti-Tumor Necrosis Factor
CUSL: Cliniques Universitaires Saint- Luc
EDI-OCT: Enhanced Depth Imaging– Optical Coherence Tomography
FA: Fluorescein Angiography
ICGA: Indocyanine Green Angiography
OCT: Optical Coherence Tomography
ROC curve: Receiver Operating Characteristic curve
SS-OCT: Swept-Source– Optical Coherence Tomography
VKH: Vogt-Koyanagi-Harada disease
